# Acoustic Propagation in a Medium with Spatially Distributed Relaxation Processes and a Possible Explanation of a Frequency Power Law Attenuation

**DOI:** 10.1142/s2591728521500122

**Published:** 2021-06-15

**Authors:** Allan D. Pierce, T. Douglas Mast

**Affiliations:** Cape Cod Institute for Science and Engineering, P. O. Box 339, East Sandwich, MA 02537, USA; Department of Biomedical Engineering, University of Cincinnati, 133 UC Bioscience Center, 3159 Eden Avenue, Cincinnati, OH 45221-0048, USA

**Keywords:** Acoustic waves, relaxation, heterogeneous media, attenuation, dispersion, phase velocity, absorption

## Abstract

A possible medium is considered with a large number of diverse but possibly similar relaxation processes. The equations for single spatially located relaxation processes of general type are developed and extended to a large spatially extended system. Each process is characterized by a relaxation time and a relaxation strength and is excited by the local pressure in an incident acoustic wave. A constant frequency model is initially assumed with complex amplitudes associated with acoustic pressure and relaxation responses. A smearing process results in the collective relaxation responses being regarded as a continuous function of relaxation times. An expression for entropy perturbation is developed in terms of the relaxation responses. The equations of fluid mechanics then lead to an expression for the pressure perturbation in terms of the dilatation and the relaxation processes. The extended result yields a time-domain wave equation for propagation through an inhomogeneous medium with distributed relaxation processes. Expressions for frequency-dependent attenuation and phase velocity are derived in which relaxation is characterized by a single function giving strength as a continuous function of relaxation time. Possible choices for this function are discussed, and it is shown that some choices lead to an attenuation varying nearly as a power of the frequency over any fixed and possibly large range of frequencies. A model set of parameters leads to good agreement with attenuation and phase velocity measurements for a suspension of human red blood cells in saline solution, as communicated to the authors by Treeby and analogous to those reported by Treeby, Zhang, Thomas, and Cox.

## 1. Introduction

A relaxation process is one where some portion or aspect of the state of a medium does not respond instantaneously to a change in the external environment. The portion does, however, tend toward equilibrium with the environment, but there is a time delay. Such a time delay generally has a characteristic time, which is referred to as a relaxation time. It has long been conjectured and/or understood that such a process can extract energy from an acoustic disturbance and place this energy elsewhere. Just where the lost energy typically goes is generally of minor interest, but that it is lost from the acoustic disturbance is often of major interest. Conjectures and theories regarding relaxation processes go back to Rayleigh,^1^ James Jeans,^2^ and Einstein.^3^ The basic theory was largely developed by Herzfeld and Rice,^4^ Kneser,^5^ and Henry.^6^ The late R. Bruce Lindsay^7^ briefly reviews this early history and gives a selection of seminal reprints in his benchmark papers volume on *Physical Acoustics*. The current literature on this topic is voluminous, and there is evidently a broad range of possible types of relaxation processes. A wide-sweeping review of the status of the subject as of 1951 is given by Markham *et al.*^8^ This review is also reprinted in Lindsay’s benchmark volume. The first author’s earlier “best shot” at a physical explanation of relaxation processes can be found in Chapter 10 of Pierce’s *Acoustics*.^9^ The account there, however, is largely limited to vibrational relaxation of diatomic molecules, which is the dominant mechanism for air. The account is somewhat similar to what was previously given by Meixner.^10^

There has long been an expectation that many multiple relaxation processes can occur in complex media. The supposition goes back at least as far as Zener’s^11^ 1948 book, *Elasticity and Anelasticity of Metals*. Zener conjectured that almost all attenuation associated with an elasticity can be attributed to relaxation processes. However, he does not give much in the way of an explicit theory to support this assertion. In later years, a number of authors have developed theories that include multiple relaxation processes. Early developments include those of Carstensen and Schwan^12^ and of Nachman *et al.*^13^ The latter included an arbitrary number of discrete relaxation processes and gave an extensive account of the theory’s consequences. A later theory (2012) of especial note is given in a paper in *Wave Motion* by Vilensky *et al.*^14^ This paper developed a somewhat comprehensive theory that included a continuous distribution of relaxation times. The literature is now very extensive, and the present authors do not here attempt to survey all applicable previous work.

This paper differs from what has previously been written in that it assumes an inhomogeneous medium and attempts to develop a theory from the perspective that the relaxation mechanisms are each spatially localized. Separate mechanisms are of two basic types, energy relaxation and volume relaxation, and each contributes to the nonequilibrium portion of the entropy of the medium. The notion of a continuous smear of relaxation processes is introduced, and the differential version of the second law of thermodynamics leads to an equation relating acoustic pressure, fluid dilatation, and the relaxation processes. The theory is admittedly somewhat intricate, but it may have some intrinsic appeal because of possible simplicity relative to what has appeared in earlier literature. The theory derived in the present paper is relatively self-contained and is developed, to the fullest abilities of the authors, from first principles and with attention to mathematical rigor and clarity. The principal idea is that the relaxations in much media can be idealized as a continuous smear, with all relaxation times being in principle possible. This leads to expressions involving integrals over relaxation time. As is explained further below, such representations can explain various seemingly-regular frequency dependences of acoustic attenuation and phase velocity dispersion.

In the latter portion of the paper, the premise is explored that the model with a continuous smear of relaxation processes may offer a physics-based explanation for the wave attenuation appearing to vary with frequency as a simple power law over a broad range of frequencies.

## 2. A Single Relaxation Process

The primary model considered in the present paper portrays the medium as containing a very large number of isolated relaxation processes. Since the processes are isolated and discrete, we could refer to the medium as a heterogeneous medium. However, over longer distance scales, of the order of an acoustic wavelength, the number of processes per unit volume may be large, and the medium may possibly be viewed as homogeneous, from the perspective of such a range of scales. Then, if the properties vary significantly over distances large compared to a wavelength, the medium can be referred to as inhomogeneous.

The relaxation processes considered here can be associated with a variety of causes. The general theory outlined further below allows one to broadly regard each process as being one of two types — energy relaxation and volume relaxation. In the former type, the energy associated with the process changes with changes in the environment, but the change tends to lag the environmental changes. For volume relaxation, the volume occupied by a distinct local cluster of matter tends to change with changes in the environment, but also with a time lag. The energy relaxation process mechanism is extensively discussed in the literature, but the volume relaxation mechanism is less discussed. To the best of the present authors’ knowledge, such a mechanism was first discussed by Leonard Hall,^15^ whose 1948 *Physical Review* paper was specifically concerned with the absorption of sound in water. The present paper’s theory is more general and may possibly be regarded as simpler. It does not attempt, however, to predict intrinsic parameters, such as relaxation times.

Let us enumerate the processes with the integer ν. The νth process is located in the vicinity of a spatial point $x_{\nu }$ and is ideally associated with two possible states, a lower state associated with some physical quantity $S_{\nu ,\text{low}}$ and an upper state associated with a quantity $S_{\nu ,\text{up}}$. The process could be supposed, in a quantum mechanical sense, to be in one state or the other. However, given that the overall system is very large and contains a large number of relaxation processes, we use a counterpart of the correspondence principle and assume that a classical limit holds in some statistical sense. The νth process is just one of many similar processes, so no great harm is done if we assume that its state can have any one of a continuum of values (as would be the case for an average of an ensemble of similar processes) between $S_{\nu ,\text{low}}$ and $S_{\nu ,\text{up}}$. We denote its instantaneous value by $S_{\nu }$.

The value $S_{\nu }$ is presumed, during equilibrium circumstances, to depend on the local pressure, which we write as $p_{o}+p^{\prime }$, where $p_{o}$ is the ambient pressure. For small disturbance pressures, we write

(2.1) $$S_{\nu }\left(p\right)=S_{\nu }\left(p_{o}\right)+{\left(\frac{dS_{\nu }}{dp}\right)}_{o}p^{\prime }.$$

(There is a possible question at this point as to whether $dS_{\nu }/dp$ is positive or negative. The sign depends on the nature of the physical quantity S and is an inherent property of the medium. Whatever the sign may be, it must be such that it results in a positive attenuation of sound. Otherwise, the medium would be unstable. This is discussed further below. The early portion of the derivation here is independent of the sign of the derivative.)

The increment in the parameter S nominally expected when the system is in equilibrium is

(2.2) $${\left(\delta S\right)}_{\nu ,eq}={\left(\frac{dS_{\nu }}{dp}\right)}_{o}p^{\prime },$$

where the derivative is evaluated at $p_{o}$.

When an acoustic wave passes by, the local increment of the pressure is $p^{\prime }$, but the value of $S_{\nu }$ may be slightly different from $S_{\nu }\left(p_{o}+p^{\prime }\right)$. Instead, the instantaneous value corresponds to a slightly different pressure, which we denote as $p_{o}+p_{\nu }$. Thus, the actual increment is

(2.3) $${\left(\delta S\right)}_{\nu ,ac}={\left(\frac{dS_{\nu }}{dp}\right)}_{o}p_{\nu },$$

where the subscript “ac” is an abbreviation for actual.

Given that the apparent pressure increment $p_{\nu }$ is not the acoustic value $p^{\prime }$, one expects it, over the course of time, to relax to $p^{\prime }$. The rate of relaxation is expected to be approximately of first order in the difference, $p_{\nu }-p^{\prime }$. Thus, one has the approximate relaxation equation

(2.4) $$\frac{dp_{\nu }}{dt}=-\frac{1}{\tau_{\nu }}\left(p_{\nu }-p^{\prime }\right),$$

where $\tau_{\nu }$ is a constant which we call the *relaxation time*.

If we consider all of the governing equations to be linear, then we can consider all disturbances to be built, in the sense of Fourier transforms, from single-constant-frequency disturbances. In this regard, we can assume each oscillatory quantity to be the real part of a complex amplitude times a time-dependent factor of $e^{-i\omega t}$. Here, we denote complex amplitudes by over-carats. Thus,

(2.5) $$p_{\nu }=Re\left\{{\overset{ˆ}{p}}_{\nu }e^{-i\omega t}\right\};\;p^{\prime }=Re\left\{\overset{ˆ}{p}e^{-i\omega t}\right\}.$$

(For brevity, the prime is omitted on ${\overset{ˆ}{p}}^{\prime }$.) For a constant-frequency disturbance of angular frequency ω, the relaxation equation implies that these complex amplitudes are related by the algebraic relation

(2.6) $${\overset{ˆ}{p}}_{\nu }=\left(\frac{1}{1\;-\;i\omega \tau_{\nu }}\right)\;\overset{ˆ}{p},\;{\overset{ˆ}{p}}_{\nu }=\overset{ˆ}{p}+\left(\frac{i\omega \tau_{\nu }}{1\;-\;i\omega \tau_{\nu }}\right)\;\overset{ˆ}{p.}$$

The second term in the latter gives the nonequilibrium part of ${\overset{ˆ}{p}}_{\nu }$.

Consequently, the complex amplitude of the sum over all relaxation processes that are in some small volume (ΔV) can be expressed as

(2.7) $$(\delta \overset{ˆ}{S})=\sum_{\nu }^{\prime }{\left(\frac{dS_{\nu }}{dp}\right)}_{o}\frac{1}{1\;-\;i\omega \tau_{\nu }}\overset{ˆ}{p,}$$

or as

(2.8) $$(\delta \overset{ˆ}{S})={\sum_{\nu }}^{\prime }\;{\left(\frac{dS_{\nu }}{dp}\right)}_{o}\overset{ˆ}{p}+{\sum_{\nu }}^{\prime }\;{\left(\frac{dS_{\nu }}{dp}\right)}_{o}\frac{i\omega \tau_{\nu }}{1\;-\;i\omega \tau_{\nu }}\overset{ˆ}{p.}$$

Here the prime on the summation sign implies that the sum is restricted to only those processes that are physically located in the volume (ΔV). We assume that this volume is small in terms of an acoustic wavelength, but large enough so that it contains a large number of relaxation processes. The indicated sum is expected to be nearly proportional to the volume.

In what follows, we ignore the equilibrium part of all mathematical expressions for relaxation processes. The equilibrium part is presumed to be factored into the general description of the medium. The ambient medium includes the equilibrium part. Thus, we write

(2.9) $$(\delta \overset{ˆ}{S}{)}_{ne}={\sum_{\nu }}^{\prime }{\left(\frac{dS_{\nu }}{dp}\right)}_{o}\frac{i\omega \tau_{\nu }}{1\;-\;i\omega \tau_{\nu }}\overset{ˆ}{p,}$$

where the subscript “ne” stands for nonequilibrium.

## 3. Integration Over Relaxation Times

To derive a suitable approximation that smears out the distinctions among various relaxation processes, we next further limit the sum to one which contains only processes whose relaxation times are within some interval (Δτ). This further restricted sum is required to have a large enough number of processes so that it will be nearly proportional to (Δτ). Thus, we write

(3.1) $${\sum_{\nu }}^{\prime \prime }{\left(\frac{dS_{\nu }}{dp}\right)}_{o}=F_{S}\left(x,\tau \right)\left(\Delta V\right)\left(\Delta \tau \right),$$

where $F_{S}(x,\tau )$ is a smeared-out function evaluated at the point x in the center of the volume and at the relaxation time in the center of the interval (Δτ).

The sum over the relaxation processes approaches an integral over relaxation times which is proportional to the small volume (ΔV). Thus, we have

(3.2) $$\frac{1}{\Delta V}{\left(\delta \overset{ˆ}{S}\right)}_{ne}=\left(\int_{o}^{\infty }\;F_{S}\left(x,\tau \right)\frac{i\omega \tau }{1\;-\;i\omega \tau }d\tau \right)\;\overset{ˆ}{p}.$$

The quantity on the left here has the units of our physical quantity S per unit volume. So we regard it as a density. It is also a complex amplitude. We denote it by the symbol ${\overset{ˆ}{w}}_{S,ne}$, where w stands for “whatever.” (The symbol s is reserved for entropy per unit mass.) So we have

(3.3) $${\overset{ˆ}{w}}_{S,ne}\left(x,\omega \right)=\left(\int_{o}^{\infty }F_{S}\left(x,\tau \right)\frac{i\omega \tau }{1\;-\;i\omega \tau }d\tau \right)\;\overset{ˆ}{p}.$$

### 3.1. Time domain version

The above equation can also be expressed in the time domain if $w_{S}(x,\omega )$ and $\overset{ˆ}{p}$ are regarded as Fourier transforms. (For brevity, in what follows, we here delete the subscript “ne.”) One writes

(3.4) $$w_{S}\left(t\right)=\int_{-\infty }^{\infty }{\overset{ˆ}{w}}_{S}\left(\omega \right)e^{-i\omega t}d\omega ,$$

(3.5) $$\overset{ˆ}{p}\left(\omega \right)=\frac{1}{2\pi }\int_{-\infty }^{\infty }p^{\prime }\left(t^{\prime }\right)e^{i\omega t^{\prime }}dt^{\prime },$$

so that

(3.6) $$w_{S}\left(x,t\right)=\frac{1}{2\pi }\int_{-\infty }^{\infty }e^{-i\omega t}\left(\int_{o}^{\infty }F_{S}\left(x,\tau \right)\frac{i\omega \tau }{1\;-\;i\omega \tau }d\tau \right)\left(\int_{-\infty }^{\infty }p^{\prime }\left(x,t^{\prime }\right)e^{i\omega t^{\prime }}dt^{\prime }\right)\;d\omega .$$

Here, ideally, one would like to interchange the order of integrations, so that one might do the ω integration first. However, the ω in the numerator poses a potential convergence problem. This is dealt with by recognition that

(3.7) $$i\omega \to -\frac{\partial }{\partial t}.$$

(The substitution is allowable because one is not integrating over t.) Making this substitution allows us to write

(3.8) $$w_{S}\left(x,t\right)=-\frac{\partial }{\partial t}\int_{o}^{\infty }\tau F_{S}\left(x,\tau \right)\int_{-\infty }^{\infty }\left(\frac{1}{2\pi }\int_{-\infty }^{\infty }\frac{e^{-i\omega \left(t-t^{\prime }\right)}}{1\;-\;i\omega \tau }d\omega \right)\;p^{\prime }\left(x,t^{\prime }\right)dt^{\prime }d\tau .$$

The indicated integral over ω that appears in the above conforms to necessary requirements so that it can be evaluated by contour integration. There is a pole at ω=−i/τ, which is in the lower half-plane. If $t<t^{\prime }$, one closes the contour with a half-circle at infinity in the upper-half plane and no pole is encircled, so the integral is zero. But, if $t>t^{\prime }$, one closes the contour in the lower-half plane and the integral is evaluated by the residue theorem. Thus,

(3.9) $$\frac{1}{2\pi }\int_{-\infty }^{\infty }\frac{e^{-i\omega \left(t-t^{\prime }\right)}}{1\;-\;i\omega \tau }d\omega \to \frac{1}{2\pi }\left[\frac{-2\pi i}{-i\tau }\right]e^{-\left(t-t^{\prime }\right)/\tau }H\left(t-t^{\prime }\right),$$

where $H\left(t-t^{\prime }\right)$ is the Heaviside step function.

The result is a time derivative of a convolution integral, given as

(3.10) $$w_{S}\left(x,t\right)=-\frac{\partial }{\partial t}\int_{-\infty }^{t}p^{\prime }\left(x,t^{\prime }\right)\left(\int_{o}^{\infty }F_{S}\left(x,\tau \right)\frac{1}{\tau }e^{-\left(t-t^{\prime }\right)/\tau }d\tau \right)\;dt^{\prime }.$$

The is nothing especially surprising about this expression. The τ-dependent factor $(1/\tau )e^{-\left(t-t^{\prime }\right)/\tau }$ is zero at τ=0, for $t>t^{\prime }$, rises to a maximum, and subsequently decreases. Knowing that the expression actually exists is nevertheless comforting, especially the prediction that the quantity $w_{S}(t)$ depends only on the past history of $p^{\prime }$ and not on its future.

[The artifice of closing the integration contour with a half circle at ∞ is possibly well-known in the mathematical physics of the past century, but possibly not so to modern readers of this paper. Give that the integrand goes as 1/ω along the real axis, the proof results from

(3.11) $$\underset{r\to \infty }{lim}\left\{\int_{o}^{2\pi }e^{-rt\;sin\;\phi }d\phi \right\}=0;\;t>0.$$

The proof fails if the integrand approaches a positive power of ω at ∞.]

## 4. Thermodynamic Considerations

There are a number of mechanisms that can cause frequency dispersion and attenuation in extended media, one of which is relaxation processes. Given the usual assertion that all mechanisms are weak, their contributions to dispersion and attenuation are additive. The present paper is concerned only with relaxation processes, so it is sufficient to use a simplified model of a medium in which the only contributor is relaxation. Consequently, we here neglect viscosity and thermal conduction. We also neglect Stokes flow around suspended particles, and we neglect sliding friction between solid particles, insofar as such are not describable as relaxation processes. Contributions of scattering, e.g. by microscopic particles, to attenuation are also neglected. With this general spirit, we regard the medium as a fluid, whose state is describable by a pressure p, a mass density ρ, an absolute temperature T, and entropy s per unit mass. It also has an internal energy u per unit mass. A related quantity is the volume v per unit mass, which is the reciprocal of ρ. These quantities can be regarded as averages over volumes large compared to the length scale of the local relaxation processes. Another quantity of interest is the mass-weighted local fluid velocity v. We here allow the possibility that all of these can depend on position x and time t. These quantities are all subject to the laws of thermodynamics and continuum mechanics.

To incorporate the nonequilibrium portions of the relaxation processes into a continuum-mechanical theory, we begin with the differential version of the second law of thermodynamics,

(4.1) Tds=du+pdv.

This equation, which appears in almost all modern textbooks of thermodynamics, asserts the existence of a state function s(u,v) which we call the entropy per unit mass. It is a function of the internal energy u per unit mass and of the volume v per unit mass. Here v=1/ρ, where ρ is the mass density, or mass per unit volume. The differential version of the equation, as stated here, gives definitions for the derivatives of this function:

(4.2) $${\left(\frac{\partial s}{\partial u}\right)}_{v}=\frac{1}{T},$$

(4.2) $${\left(\frac{\partial s}{\partial v}\right)}_{u}=\frac{p}{T},$$

where T is the absolute temperature and p is the total pressure.

We conceive of the nonequilibrium portions of relaxation processes as affecting either the internal energy u or the specific volume v, and the contributions are additive. Thus, we write

(4.4) $$u=u_{\text{eq}}+u_{\text{relax,ne}};\;v=v_{\text{eq}}+v_{\text{relax,ne}},$$

where the subscript “eq” is here an abbreviation for “equilibrium.” With the neglect of thermal conductivity, the entropy for a (possibly moving) media particle is unchanged by an acoustic disturbance in the absence of relaxation. Thus, the change in entropy to first order in relaxation amplitudes is

(4.5) $$\Delta s=\frac{1}{T_{o}}u_{relax,ne}+\frac{p_{o}}{T_{o}}v_{relax,ne}.$$

In the usual scheme of thermodynamics, pressure can be regarded as a function of any two thermodynamic variables, which we here choose as entropy and density. We can write the fluctuation in pressure via the use of partial derivatives as

(4.6) $$\delta p={\left(\frac{\partial p}{\partial \rho }\right)}_{s}\delta \rho +{\left(\frac{\partial p}{\partial s}\right)}_{\rho }\delta s.$$

The first partial derivative is

(4.7) $${\left(\frac{\partial p}{\partial \rho }\right)}_{s}=c^{2},$$

where c is the equilibrium speed of sound. The second partial derivative requires some analytical manipulation to be expressed in terms of standard thermodynamic coefficients. The appropriate derivation begins with

(4.8) $${\left(\frac{\partial p}{\partial s}\right)}_{\rho }=-\frac{{\left(\frac{\partial \rho }{\partial s}\right)}_{p}}{{\left(\frac{\partial \rho }{\partial p}\right)}_{s}}=-\frac{{\left(\frac{\partial v}{\partial s}\right)}_{p}}{{\left(\frac{\partial v}{\partial p}\right)}_{s}}=-\frac{{\left(\frac{\partial T}{\partial p}\right)}_{s}}{{\left(\frac{\partial v}{\partial p}\right)}_{s}},$$

where

(4.9) $${\left(\frac{\partial v}{\partial s}\right)}_{p}={\left(\frac{\partial T}{\partial p}\right)}_{s}$$

is one of the Maxwell relations of thermodynamics. It follows from

(4.10) $$d\left(u+pv\right)=Tds-pdv+pdv+vdp=Tds+vdp,$$

with the equivalence of the two expressions for the second derivative of the quantity u+pv, regarded as a function of s and p. One can also write

(4.11) $${\left(\frac{\partial T}{\partial p}\right)}_{s}=-\frac{{\left(\frac{\partial T}{\partial s}\right)}_{p}}{{\left(\frac{\partial p}{\partial s}\right)}_{T}}=\frac{{\left(\frac{\partial T}{\partial s}\right)}_{p}}{{\left(\frac{\partial T}{\partial v}\right)}_{p}},$$

where

(4.12) $${\left(\frac{\partial s}{\partial p}\right)}_{T}=-{\left(\frac{\partial v}{\partial T}\right)}_{p}$$

is another of the Maxwell relations. Another required mathematical identity is

(4.13) $${\left(\frac{\partial v}{\partial p}\right)}_{s}=-\frac{1}{\rho^{2}}{\left(\frac{\partial \rho }{\partial p}\right)}_{s}.$$

The analysis above yields the thermodynamic identity

(4.14) $${\left(\frac{\partial p}{\partial s}\right)}_{\rho }=\rho^{2}{\left(\frac{\partial p}{\partial \rho }\right)}_{s}\frac{{\left(\frac{\partial T}{\partial s}\right)}_{p}}{{\left(\frac{\partial T}{\partial v}\right)}_{p}}.$$

This form is expressible in terms of standard thermodynamic coefficients:

(4.15) $$c^{2}={\left(\frac{\partial p}{\partial \rho }\right)}_{s},$$

(4.16) $$c_{p}=T{\left(\frac{\partial s}{\partial T}\right)}_{p},$$

(4.17) $$\beta =\frac{1}{v}{\left(\frac{\partial v}{\partial T}\right)}_{p},$$

where c is the speed of sound, $c_{p}$ is the specific heat at constant pressure, and β is the coefficient of volume expansion. With the use of these symbols, one has

(4.18) $${\left(\frac{\partial p}{\partial s}\right)}_{\rho }=\frac{\rho c^{2}\beta T}{c_{p}}.$$

Thus, our differential relation for the pressure fluctuation can be written as

(4.19) $$\delta p=c^{2}\delta \rho +{\left(\frac{\rho c^{2}\beta T}{c_{p}}\right)}_{o}\left[\frac{1}{T_{o}}u_{\text{relax},\text{ne}}+\frac{p_{o}}{T_{o}}v_{\text{relax,}\;\text{ne}}\right],$$

where the subscript “o” implies the quantity is evaluated at the ambient state.

The above relation applies to a fixed “particle” of a stationary medium, and some care should be exercised if the particle is moving and if the ambient medium is inhomogeneous. To account for this, the state function increments should be replaced by total time derivatives, so that we have

(4.20) $$\frac{Dp}{Dt}=c^{2}\frac{D\rho }{Dt}+{\left(\frac{\rho c^{2}\beta T}{c_{p}}\right)}_{o}\left[\frac{1}{T_{o}}\frac{D}{Dt}u_{\text{relax},\text{ne}}+\frac{p_{o}}{T_{o}}\frac{D}{Dt}v_{\text{relax},\text{ne}}\right],$$

where the total time derivative operator, for a small amplitude disturbance, is given by (for example)

(4.21) $$\frac{Dp}{Dt}=\frac{\partial p^{\prime }}{\partial t}+v^{\prime }\cdot \nabla p_{o}.$$

In the current case, the only ambient state function appearing here that is expected to vary appreciably with displacement is the ambient density $\rho_{o}$. The governing equations, with the neglect of gravity, require $p_{o}$ to be constant. Also, the relaxation quantities are small, so any terms involving spatial gradients of their ambient values are negligible. With regard to the total time derivative of the density, the conservation of mass equation requires, in the linear approximation,

(4.22) $$\frac{D\rho }{Dt}+\rho_{o}\nabla \cdot v^{\prime }=0.$$

Consequently, Eq. (4.20) becomes

(4.23) $$\frac{\partial p^{\prime }}{\partial t}=-\rho_{o}c^{2}\nabla \cdot v^{\prime }+{\left(\frac{\rho c^{2}\beta T}{c_{p}}\right)}_{o}\left[\frac{1}{T_{o}}\frac{\partial }{\partial t}u_{\text{relax,ne}}+\frac{p_{o}}{T_{o}}\frac{\partial }{\partial t}v_{\text{relax,ne}}\right].$$

The above result should be applicable to the situation where an acoustic wave is propagating through the medium. The disturbance can be made up of many frequencies, but the governing equations are regarded as linear, so it is sufficient to consider a single frequency component, and assume $e^{-i\omega t}$ time dependence. The complex amplitudes are consequently related as

(4.24) $$\overset{ˆ}{p}=\frac{1}{i\omega }\rho_{o}c^{2}\nabla \cdot \overset{ˆ}{v}+{\left(\frac{\rho c^{2}\beta T}{c_{p}}\right)}_{o}\left[\frac{1}{T_{o}}{\overset{ˆ}{u}}_{\text{relax,ne}}+\frac{p_{o}}{T_{o}}{\overset{ˆ}{v}}_{\text{relax,ne}}\right].$$

[In the event the medium is homogeneous, so that $\rho_{o}$ is independent of position, the conservation of mass relation allows the above to be replaced by

(4.25) $$\overset{ˆ}{p}=c^{2}\overset{ˆ}{\rho }+{\left(\frac{\rho c^{2}\beta T}{c_{p}}\right)}_{o}\left[\frac{1}{T_{o}}{\overset{ˆ}{u}}_{\text{relax,ne}}+\frac{p_{o}}{T_{o}}{\overset{ˆ}{v}}_{\text{relax,ne}}\right].$$

The discussion below proceeds, however, as if the medium were inhomogeneous.]

The analysis in the preceding section yields relaxation quantities that are per unit volume. Since relaxation processes do not change the mass density, we need only multiply by $\left(1/\rho_{o}\right)$ to obtain the relaxation quantity per unit mass. Thus, one has

(4.26) $${\overset{ˆ}{u}}_{\text{relax,ne}}=\frac{1}{\rho_{o}}{\overset{ˆ}{w}}_{E,\text{ne}}=\frac{1}{\rho_{o}}\left(\int_{o}^{\infty }F_{E}(x,\tau )\frac{i\omega \tau }{1\;-\;i\omega \tau }d\tau \right)\;\overset{ˆ}{p,}$$

(4.27) $${\overset{ˆ}{v}}_{\text{relax,ne}}=\frac{1}{\rho_{o}}{\overset{ˆ}{w}}_{V,\text{ne}}=\frac{1}{\rho_{o}}\left(\int_{o}^{\infty }F_{V}(x,\tau )\frac{i\omega \tau }{1\;-\;i\omega \tau }d\tau \right)\;\overset{ˆ}{p}.$$

These two relations allow us to write the pressure-dilatation relation, Eq. (4.24), as

(4.28) $$\overset{ˆ}{p}=\frac{1}{i\omega }\rho_{o}c^{2}\nabla \cdot \overset{ˆ}{v}-K_{relax}(\omega ,x)\overset{ˆ}{p,}$$

where

(4.29) $$K_{relax}\left(\omega ,x\right)=-{\left(\frac{c^{2}\beta }{c_{p}}\right)}_{o}\left(\int_{o}^{\infty }\left[F_{E}\left(x,\tau \right)+p_{o}F_{V}\left(x,\tau \right)\right]\frac{i\omega \tau }{1\;-\;i\omega \tau }d\tau \right).$$

With an additional abbreviation, this can be rewritten as

(4.30) $$K_{relax}\left(\omega ,x\right)=\int_{o}^{\infty }\frac{i\omega \tau G\left(x,\tau \right)}{1\;-\;i\omega \tau }d\tau ,$$

where

(4.31) $$G(x,\tau )=-{\left(\frac{c^{2}\beta }{c_{p}}\right)}_{o}\left[F_{E}(x,\tau )+p_{o}F_{V}(x,\tau )\right].$$

The insertion of minus signs here is with some hindsight into the derivations that follow below for the acoustic attenuation, which should be positive. Consequently, $F_{E}$ and $F_{V}$ should be negative.

[A slight reality check can be made on the physical dimensions that appear here. The quantity $F_{E}$ has the units of energy divided by pressure, divided by volume times time, or of 1 over time. The quantity $c^{2}\beta /c_{p}$ has the units of length squared over time squared divided by energy over mass, so it is dimensionless. All this is consistent with $K_{\text{relax}}$ being dimensionless. An analogous argument holds for the $p_{o}F_{V}$ term: $F_{V}$ has units of 1 divided by pressure times time.]

### 4.1. Transient version of pressure-dilatation relation

To derive a transient version of Eq. (4.28), one first multiplies both sides by $-i\omega e^{-i\omega t}$ and then integrates over ω from −∞ to ∞. Given the definition of the Fourier transform, doing this yields

(4.32) $$\frac{\partial p}{\partial t}=-\rho_{o}c^{2}\nabla \cdot v+\int_{-\infty }^{\infty }i\omega K_{relax}\left(\omega ,x\right)\overset{ˆ}{p}e^{-i\omega t}d\omega .$$

For the second term on the right, we insert the inverse Fourier transform

(4.33) $$\overset{ˆ}{p}=\frac{1}{2\pi }\int_{-\infty }^{\infty }p\left(t^{\prime }\right)e^{i\omega t^{\prime }}dt^{\prime }.$$

The second term on the right of Eq. (4.32) consequently can be written as

(4.34) $$\frac{1}{2\pi }\int_{-\infty }^{\infty }\int_{-\infty }^{\infty }p\left(t^{\prime }\right)\left(-\omega^{2}\right)e^{-i\omega \left(t-t^{\prime }\right)}\left(\int_{0}^{\infty }\frac{\tau G(x,\;\tau )}{1\;-\;i\omega \tau }d\tau \right)\;dt^{\prime }d\omega .$$

Here one is tempted to interchange the orders of integration and do the ω integration first. But the resulting integral over ω does not converge because of the $\omega^{2}$ in the numerator. To circumvent this problem, one notices that one is not integrating over t and that

(4.35) $$-\omega^{2}e^{-i\omega \left(t-t^{\prime }\right)}\to \;\frac{\partial^{2}}{\partial t^{2}}e^{-i\omega \left(t-t^{\prime }\right)}.$$

One can accordingly move the time derivative outside the integral, so the second term on the right of Eq. (4.32) becomes

(4.36) $$\frac{1}{2\pi }\frac{\partial^{2}}{\partial t^{2}}\int_{-\infty }^{\infty }\int_{-\infty }^{\infty }p\left(t^{\prime }\right)e^{-i\omega \left(t-t^{\prime }\right)}\left(\int_{o}^{\infty }\frac{\tau G(x,\;\tau )}{1\;-\;i\omega \tau }d\tau \right)\;dt^{\prime }d\omega .$$

Now we can safely interchange the order of integration and we find the integral

(4.37) $$J\left(\left[t-t^{\prime }\right]/\tau \right)=\int_{-\infty }^{\infty }\frac{\tau e^{-i\omega \left(t-t^{\prime }\right)}}{1\;-\;i\omega \tau }d\omega .$$

Here we again have an integral that can be evaluated by the residue theorem. There is a pole in the lower half-plane at ω=−i/τ, and one can close the contour when $t>t^{\prime }$ by a half circle in the lower half-plane at ∞. The residue theorem consequently gives

(4.38) $$J\left(\left[t-t^{\prime }\right]/\tau \right)=\left(-2\pi i\right)\frac{\tau }{-i\tau }e^{-\frac{\left(t-t^{\prime }\right)}{\tau }}H\left(t-t^{\prime }\right)=2\pi e^{-\frac{\left(t-t^{\prime }\right)}{\tau }}H\left(t-t^{\prime }\right),$$

where $H\left(t-t^{\prime }\right)$ is the unit step function. The latter is taken into account by setting the upper limit of the $t^{\prime }$ integration to t.

The mathematical steps described above lead to a transient relation between pressure and dilatation having the form

(4.39) $$\frac{\partial p}{\partial t}=-\rho_{o}c^{2}\nabla \cdot v\;+\;\frac{\partial^{2}}{\partial t^{2}}\int_{-\infty }^{t}\Psi \left(x,t-t^{\prime }\right)p\left(t^{\prime }\right)dt^{\prime },$$

where

(4.40) $$\Psi \left(x,t-t^{\prime }\right)=\int_{o}^{\infty }G\left(x,\tau \right)e^{-\left(t-t^{\prime }\right)/\tau }d\tau .$$

## 5. Transient Wave Equation

The linearized equations of fluid dynamics yield the acoustics equations: the Euler equation and the conservation of mass,

(5.1) $$\rho_{o}\frac{\partial v}{\partial t}=-\nabla p,$$

(5.2) $$\frac{\partial \rho }{\partial t}+\nabla \cdot \left(\rho_{o}v\right)=0,$$

and from these, one derives

(5.3) $$\frac{\partial \rho }{\partial t}+v\cdot \nabla \rho_{o}+\rho_{o}\nabla \cdot v=0,$$

(5.4) $$\frac{\partial }{\partial t}\nabla \cdot v=-\nabla \cdot \left(\frac{1}{\rho_{o}}\nabla p\right),$$

(5.5) $$\frac{\partial^{2}\rho }{\partial t^{2}}=\nabla^{2}p.$$

[Note that these equations do not involve a speed of sound and they do not, by themselves, yield a wave equation or a Helmholtz equation.] These equations apply to an inhomogeneous medium in which the ambient density $\rho_{o}$, the sound speed c, and other medium properties are functions of position.

The formulation as developed in the previous section with the equations given above leads to a wave equation for the acoustic portion of the pressure. If one takes the time derivative of both sides of Eq. (4.39) and then eliminates the time derivative of the divergence of the velocity using Eq. (5.4), one obtains the equation

(5.6) $$\frac{\partial^{2}p}{\partial t^{2}}=\rho_{o}c^{2}\nabla \cdot \left(\frac{1}{\rho_{o}}\nabla p\right)+\frac{\partial^{3}}{\partial t^{3}}\int_{-\infty }^{t}\Psi \left(t-t^{\prime }\right)p\left(t^{\prime }\right)dt^{\prime },$$

which can be written in a slightly more familiar form as

(5.7) $$\rho c^{2}\nabla \cdot \left(\frac{1}{\rho }\nabla p\right)-\frac{\partial^{2}p}{\partial t^{2}}=-\frac{\partial^{3}}{\partial t^{3}}\int_{-\infty }^{t}\Psi \left(x,t-t^{\prime }\right)p\left(t^{\prime }\right)dt^{\prime }.$$

Here, for simplicity, the primes on the acoustic pressure perturbations have been dropped. We also drop the subscript “o” on $\rho_{o}$. These depend on position as well as on time. The influence of the relaxation processes is included in the function $\Psi \left(x,t-t^{\prime }\right)$, which is an integral involving the function G(τ,x). The integration limits in Eq. (5.7) ensure that causality is maintained, and thus, that the Kramers–Kronig relations between attenuation and phase velocity are satisfied.

### 5.1. Other wave equations

This wave equation derived here as Eq. (5.7) is somewhat similar to one proposed somewhat earlier by Szabo^16^ in a paper published in 1994, in that Szabo also proposed a second-order wave equation which had a convolution term in his wave equation. Szabo’s equation was intended to apply when the attenuation obeyed a frequency-power law and had the form

(5.8) $$\nabla^{2}p-\frac{1}{c_{o}^{2}}\frac{\partial^{2}p}{\partial t^{2}}+L_{\gamma }\;*\;p=0.$$

Here the “*” implies a convolution operator. This operator is presumably that which is defined in Bracewell’s book^17^ as

(5.9) $$f\left(t\right)*g\left(t\right)=\int_{-\infty }^{\infty }f\left(t^{\prime }\right)g\left(t-t^{\prime }\right)dt^{\prime }.$$

The function $L_{\gamma }$ is somewhat difficult to describe, but is intended to be such that the derived plane wave attenuation is proportional to a power of ω. Szabo’s paper mentions relaxation, but does not use such in the derivation of his wave equation.

What is proffered as improvements over Szabo’s equation is given in a 2003 paper by Chen and Holm.^18^ One candidate wave equation is given by (Eq. (10))

(5.10) $$\nabla^{2}p-\frac{1}{c_{o}^{2}}\frac{\partial^{2}p}{\partial t^{2}}-\frac{2\alpha_{o}}{c_{o}}S_{y}(p)=0,$$

where the functional $S_{y}(p)$ is defined piece-wise for various ranges of the attenuation exponent y in Eq. (14). Convolution certainly enters into the Chen and Holm wave equations, but the formulation has considerable (perhaps necessary) complexity, in contrast to the wave equation of the present paper.

Numerical solution of such wave equations, incorporating relaxation processes via a convolution with the acoustic pressure or its derivatives, is feasible. For example, Tabei *et al*.^19^ numerically solved first-order wave propagation equations for an inhomogeneous medium with a finite number of relaxation processes, introducing a state variable to account for a similar convolution term. Such numerical solutions may be facilitated by analytic evaluation of the function Ψ from its definition in Eq. (4.40), which is feasible for certain choices of the weighting function G(x,τ). Tabei *et al*. also showed that linear dependence of absorption on frequency could be approximated over the frequency range of 0–5 MHz by incorporation of two discrete relaxation processes.

## 6. Dispersion Relation

To derive a dispersion relation, connecting complex wave number k and angular frequency ω, one assumes the medium is homogeneous and postulates plane wave expressions,

(6.1) $$\overset{ˆ}{p}=Pe^{-i\omega t+ikx};\;\overset{ˆ}{\rho }=Re^{-i\omega t+ikx},$$

where P and R are constants. Equation (4.28), with the conservation of mass relation for a homogeneous medium, yields

(6.2) $$\left(1+K_{\text{relax}}\right)P=c^{2}R,$$

while Eq. (5.5) yields

(6.3) $$k^{2}=\frac{R}{P}\omega^{2},$$

and these two equations yield

(6.4) $$k^{2}=\frac{\omega^{2}}{c^{2}}\left[1+K_{\text{relax}}\right].$$

The magnitude of the quantity $K_{\text{relax}}$ is assumed to be much less than unity (weak dispersion), so the square root of the above yields

(6.5) $$k=\frac{\omega }{c}\left[1+\frac{1}{2}K_{relax}\right].$$

We separate this into real and imaginary parts and set

(6.6) $$k=\frac{\omega }{v_{ph}}+i\alpha ,$$

where $v_{ph}$ is the phase velocity and α is the attenuation coefficient, and identify

(6.7) $$\alpha =\frac{\omega }{2c}Im\left\{K_{\text{relax}}\right\},$$

$$\frac{1}{v_{\text{ph}}}=\frac{1}{c}\left[1+\frac{1}{2}Re\left\{K_{\text{relax}}\right\}\right],$$

or

(6.9) $$v_{ph}=c-\frac{c}{2}Re\left\{K_{relax}\right\}.$$

With the integral relations inserted, these two relations become

(6.10) $$\alpha =\frac{\omega }{2c}\int_{o}^{\infty }\frac{\omega \tau G(\tau )}{1\;+\;{\left(\omega \tau \right)}^{2}}d\tau ,$$

(6.11) $$v_{ph}=c+\frac{c}{2}\int_{o}^{\infty }\frac{{\left(\omega \tau \right)}^{2}G(\tau )}{1\;+\;{\left(\omega \tau \right)}^{2}}d\tau .$$

The former expression guarantees that the attenuation will be positive if the quantity G is positive. This justifies the choices for signs that was made in a previous section. The expression for the phase velocity predicts that c will be the equilibrium sound speed in the limit of zero frequency.

## 7. Connection with Nachman, Smith, and Waag

The formulation developed in the previous sections has strong similarities to those developed in a 1995 paper by Nachman *et al*.^13^ Some of these similarities are pointed out here.

A principal result in the cited paper is Eq. (37), which reads

(7.1) $$\kappa \left(\omega \right)=\frac{1}{c^{2}\rho_{o}}+\sum_{\nu }\kappa_{\nu }\frac{i\omega }{-i\omega \;+\;\frac{1}{\tau_{\nu }}},$$

where κ(ω) is stated to be a generalized compressibility. Our interpretation of this quantity is that in the absence of relaxation, it is just the reciprocal of the bulk modulus. The above-stated equation for the generalized compressibility can be compared with Eq. (48), here restated as

(7.2) $$\overset{ˆ}{p}=\frac{1}{i\omega }\rho_{o}c^{2}\nabla \cdot \overset{ˆ}{v}-\left(\int_{o}^{\infty }\frac{i\omega \tau G(x,\;\tau )}{1\;-\;i\omega \tau }d\tau \right)\;\overset{ˆ}{p,}$$

The latter can be rewritten as

(7.3) $$\frac{1}{i\omega }\frac{\nabla \cdot \overset{ˆ}{v}}{\overset{ˆ}{p}}=\frac{1}{\rho_{o}c^{2}}+\frac{1}{\rho_{o}c^{2}}\int_{o}^{\infty }\frac{i\omega \tau G(x,\;\tau )}{1\;-\;i\omega \tau }d\tau .$$

The generalized compressibility is appropriately identified as

(7.4) $$\kappa (\omega )=\frac{1}{i\omega }\frac{\nabla \cdot \overset{ˆ}{v}}{\overset{ˆ}{p}},$$

which is the ratio of the complex amplitude of the negative of the dilatation to that of the acoustic portion of the pressure. Nachman and his colleagues assumed a discrete set of relaxation times, but the generalization to a continuous smear is evident from a comparison of the two expressions. The connection is given by

(7.5) $$\kappa_{\nu }\Delta \nu \to \frac{G(\tau )}{\rho_{o}c^{2}}\Delta \tau .$$

Another evident similarity comes from comparison of the expressions for the attenuation. Nachman and his colleagues, in Eq. (43), give

(7.6) $$\alpha =\frac{\omega \rho_{o}v_{ph}}{2}\sum_{\nu }\frac{\kappa_{\nu }\tau_{\nu }\omega }{1\;+\;\tau_{\nu }^{2}\omega^{2}}.$$

The formulation in the present paper gives, Eq. (6.10),

(7.7) $$\alpha =\frac{\omega }{2c}\int_{o}^{\infty }\frac{\omega \tau G(\tau )}{1\;+\;{\left(\omega \tau \right)}^{2}}d\tau .$$

The present paper makes a small dispersion approximation, so we would have set $v_{ph}\to c$ in the attenuation expression. We also have the correspondence going from sum over ν to integral over τ. Thus, we have

(7.8) $$\alpha \to \frac{\omega \rho_{o}c}{2\rho_{o}c^{2}}\int_{o}^{\infty }\frac{G(\tau )\tau \omega }{1\;+\;\tau^{2}\omega^{2}}d\tau ,$$

which is the same as appears in the present paper.

Other comparisons of the earlier paper by Nachman *et al*. with the present paper can be derived, and one can rightly argue that much of the principal ideas of the present paper were already present in the earlier paper and in perhaps other papers. However, the present authors argue that a fresh account is needed to obtain a coherent exposition of the overall theory. They also hope that many readers will find the theoretical development here to be somewhat more palatable.

## 8. Frequency Dependences

The integration over relaxation time τ that appears in Eq. (6.10) for the frequency-dependent attenuation depends critically on the functional form of the function G(τ). At this point, we know relatively little about this function, but we can nevertheless study its relationship to α(ω) and $v_{ph}(\omega )$. With regard to the attenuation, at the outset, we can state that in the limit of low frequencies, after doing a power series expansion of the integrand,

(8.1) $$\alpha \to \frac{\omega^{2}}{2c}\int_{o}^{\infty }\tau G\left(\tau \right)d\tau -\frac{\omega^{4}}{2c}\int_{o}^{\infty }\tau^{3}G\left(\tau \right)d\tau .$$

[Here it is implicitly assumed that all of the indicated definite integrals exist. Such is not explicitly guaranteed, but we argue that the function G(τ) should be finite or zero in the limit of τ→0. Also, it is presumed to be negligibly small beyond a certain upper limit. The models proposed further below conform to these expectations.] The attenuation consequently goes initially as being proportional to $\omega^{2}$, but the tendency is to go as something slower at finite frequencies. An upper limit for the transition frequency is obtained by equating the two terms in the above, which yields

(8.2) $$\omega_{trans}^{2}=\frac{\int_{o}^{\infty }\tau G(\tau )d\tau }{\int_{o}^{\infty }\tau^{3}G(\tau )d\tau }.$$

In the same low frequency limit, the phase velocity goes as

(8.3) $$v_{ph}\to c+\frac{\omega^{2}c}{2}\int_{o}^{\infty }\tau^{2}G\left(\tau \right)d\tau ,$$

so it equals the ambient sound speed c at zero frequency and initially increases as the square of the frequency.

In the high frequency limit, the attenuation approaches a constant,

(8.4) $$\alpha \to \frac{1}{2c}\int_{o}^{\infty }\frac{G}{\tau }d\tau -\frac{1}{2c\omega^{2}}\int_{o}^{\infty }\frac{G}{\tau^{3}}d\tau ,$$

and the phase velocity also approaches a constant,

(8.5) $$v_{ph}\to c+\frac{c}{2}\int_{o}^{\infty }G\left(\tau \right)d\tau .$$

[Here again, there is an implied assumption that all of the indicated definite integrals exist. This is not necessarily so, but is so for the models proposed further below.]

If the formulation had included shear viscosity, such would have eventually dominated at high frequency, and the attenuation would have varied asymptotically as $\omega^{2}$.

For intermediate frequencies, there is some evidence for some media that the attenuation varies over a wide range of frequencies as a simple power law, with $\alpha \propto \omega^{a}$ for some exponent a. For example, Duck^20^ summarizes a wide literature of measured ultrasonic attenuation in biological tissue, in which attenuation was found to depend on a power of frequency within the range 1<a<2. Duck also summarizes measurements of the coefficient $\alpha_{o}$ from multiple studies in which the exponent a was assumed equal to 1.

Linear frequency dependence, with a=1, is also commonly reported for a large variety of natural media. One may suppose that this frequency dependence could be “explained” if G(τ) itself varied over a wide range of relaxation time τ also as a power law. If one considers the indefinite integral

(8.6) $$I=\int \;\frac{\omega^{2}\tau G(\tau )}{1\;+\;{\left(\omega \tau \right)}^{2}}d\tau ,$$

and sets

(8.7) $$G\left(\tau \right)=\frac{K_{a}}{\tau^{a}},$$

and then changes the variable of integration to

(8.8) ξ=ωτ,

then

(8.9) $$I\to K_{a}\omega^{a}\int \;\frac{\xi }{\xi^{a}\left(1\;+\;\xi^{2}\right)}d\xi .$$

This suggests that should the function G(τ) vary as $1/\tau^{a}$ over a wide range of relaxation times, then the attenuation would vary as $\omega^{a}$ over a wide range.

To explore the above assertion, in the remainder of this paper, we consider the special case where G=0 outside the range $\tau_{1}<\tau <\tau_{2}$. We first consider the relaxation strength for the relaxation-time region in between to vary inversely as the relaxation time (a=1), so we take

(8.10) $$G=\frac{K_{1}}{\tau }.$$

[Further below, the general case of a≠1 is considered. The case of a=1 is considered first because the mathematical expressions are somewhat simpler.]

For this special case of a=1, the integral expressions, Eqs. (6.10) and (6.11), for attenuation and phase velocity become

(8.11) $$\alpha =\frac{\omega }{2c}K_{1}\int_{\tau_{1}}^{\tau_{2}}\frac{\omega }{1\;+\;{\left(\omega \tau \right)}^{2}}d\tau ,$$

(8.12) $$v_{ph}=c+\frac{c}{2}K_{1}\int_{\tau_{1}}^{\tau_{2}}\frac{\omega^{2}\tau }{1\;+\;{\left(\omega \tau \right)}^{2}}d\tau .$$

The two integrals that appear here are of standard forms and the integrations are readily performed. Thus, we have

(8.13) $$\alpha =\frac{\omega }{2c}K_{1}\left[{tan}^{-1}\left(\omega \tau_{2}\right)-{tan}^{-1}\left(\omega \tau_{1}\right)\right],$$

(8.14) $$v_{ph}=c+\frac{c}{2}K_{1}\frac{1}{2}[ln(1+{\left(\omega \tau_{2}\right)}^{2})-ln(1+{\left(\omega \tau_{1}\right)}^{2})].$$

These two expressions have the expected behavior within low-frequency, mid-frequency, and high-frequency regimes. These follow from asymptotic expressions for the arc-tangent and the logarithm. For low frequencies, where $\omega \ll 1/\tau_{1}$ and $\omega \ll 1/\tau_{2}$, one has

(8.15) $$\alpha \approx \frac{\omega^{2}}{2c}K_{1}\left[\tau_{2}-\tau_{1}\right],$$

(8.16) $$v_{ph}\approx c+\frac{c}{2}K_{1}\frac{1}{2}\omega^{2}[\tau_{2}^{2}-\tau_{1}^{2}].$$

In the mid-frequency region, where

(8.17) $$\frac{1}{\tau_{2}}\ll \omega \ll \frac{1}{\tau_{1}},$$

one has

(8.18) $$\alpha \approx \frac{\omega }{2c}K_{1}\frac{\pi }{2},$$

(8.19) $$v_{ph}\approx c+\frac{c}{2}K_{1}\;ln\left(\omega \tau_{2}\right).$$

Then, in the high frequency limit, where $\omega \tau_{2}\gg 1$, one has

(8.20) $$\alpha \approx \frac{1}{2c}K_{1}\left[\frac{1}{\tau_{1}}-\frac{1}{\tau_{2}}\right],$$

(8.21) $$v_{ph}=c+\frac{c}{2}K_{1}\;ln\left(\frac{\tau_{2}}{\tau_{1}}\right).$$

The transitions between these three cases can be examined, for the attenuation, by writing our expression in Eq. (8.13) in the form

(8.22) $$\alpha =A\xi \left[{tan}^{-1}(M\xi )-{tan}^{-1}(\xi )\right],$$

where $\xi =\omega \tau_{1},\;M=\tau_{2}/\tau_{1}$, and $A=K_{1}/\left(2c\tau_{1}\right)$, where M is somewhat larger than 1. The three limiting expressions correspond to

(8.23) $$\frac{\alpha }{A}\approx (M-1)\xi^{2};\;M\xi \ll 1,$$

(8.24) $$\frac{\alpha }{A}\approx \xi \frac{\pi }{2};\;M\xi \gg 1,\;\xi \ll 1,$$

(8.25) $$\frac{\alpha }{A}\approx 1-\frac{1}{M};\;M\xi \gg 1,\;\xi \gg 1.$$

One can easily sketch the general shape of the attenuation curve by drawing each of these “curves” and then finding their intersections, which give the transition frequencies. The low frequency regime is described by a parabola and the mid-frequency regime is described by a straight line passing through the origin. These lines intersect at

(8.26) $$\xi_{1,2}=\frac{\frac{\pi }{2}}{M\;-\;1};\;\omega_{1,2}=\frac{\frac{\pi }{2}}{\tau_{2}\;-\;\tau_{1}}.$$

The sloped straight line corresponding to the mid-frequency region intersects the horizontal straight line corresponding to the high-frequency region when

(8.27) $$\xi_{2,3}=\frac{2}{\pi }\left(1-\frac{\tau_{1}}{\tau_{2}}\right);\;\omega_{2,3}=\frac{2}{\pi \tau_{1}}\left(1-\frac{\tau_{1}}{\tau_{2}}\right).$$

Thus, given $\tau_{2}\gg \tau_{1}$, the lower transition frequency goes as $1/\tau_{2}$ and the upper transition frequency goes as $1/\tau_{1}$.

If one desires to plot the attenuation defined by Eq. (8.22), it is first convenient to determine just how far one should plot ξ until the function arrives to within, say, 90% of its asymptotic value. To this purpose, one sets

(8.28) $$M\xi =\tan\left(\frac{\pi }{2}-\epsilon \right)=\frac{\cos\epsilon }{\sin\epsilon }\approx \frac{1}{\epsilon \;+\;\left(\frac{1}{3}\right)\;\epsilon^{3}}.$$

Thus,

(8.29) $$\epsilon +\left(\frac{1}{3}\right)\epsilon^{3}\approx \frac{1}{M\xi };\;\epsilon \approx \frac{1}{M\xi }-\left(\frac{1}{3}\right)\frac{1}{{\left(M\xi \right)}^{3}}$$

and this yields

(8.30) $$\tan^{-1}\left(M\xi \right)\approx \frac{\pi }{2}-\frac{1}{M\xi }+\frac{1}{3}\frac{1}{{\left(M\xi \right)}^{3}}.$$

The revised high frequency approximation is then

(8.31) $$\frac{\alpha }{A}\approx \left(1-\frac{1}{M}\right)-\frac{1}{3}\left(1-\frac{1}{M^{3}}\right)\frac{1}{\xi^{2}}.$$

Consequently, the function reaches 90% of its asymptotic value when

(8.32) $$\xi^{2}\approx 3\left(1+\frac{1}{M^{2}}\right).$$

**Fig. 1.**
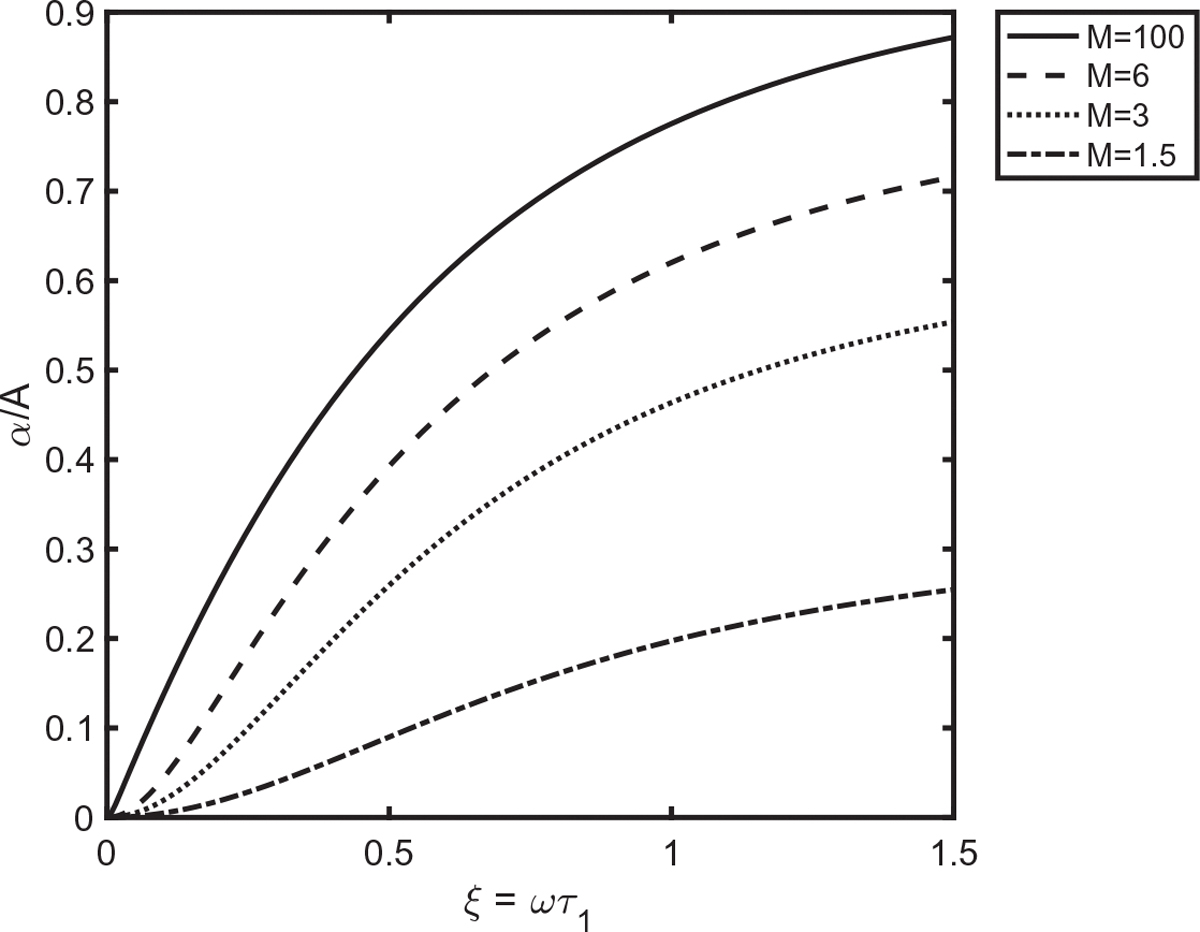
Plots of normalized attenuation versus normalized frequency for multiple values of the parameter M.

Figure 1 gives plots of α/A versus ξ for M=1.5,M=3,M=6, and M=100 for 0<ξ<1.5. The parabolic behavior at small ξ is evident in the curves for the smaller values of M, but barely detectable for M=100. The upper limit of ξ=1.5 is sufficient for one to sense the limit where the function approaches a constant at large ξ.

One can similarly write the phase velocity versus frequency for this special case of a=1 in a dimensionless form as

(8.33) $$\frac{v_{\text{phase}}}{c}-1=B\left[ln\left(1+M^{2}\xi^{2}\right)-ln\left(1+\xi^{2}\right)\right],$$

where $B=K_{1}/4$. For small ξ, this goes as

(8.34) $$\frac{v_{\text{phase}}}{c}-1=B\left(M^{2}-1\right)\xi^{2},$$

and for large ξ, it goes as

(8.35) $$\frac{v_{\text{phase}}}{c}-1=B\;ln\;M^{2}.$$

In the intermediate range, it goes as

(8.36) $$\frac{v_{\text{phase}}}{c}-1=B\;\ln\left(M^{2}\xi^{2}\right).$$

**Fig. 2.**
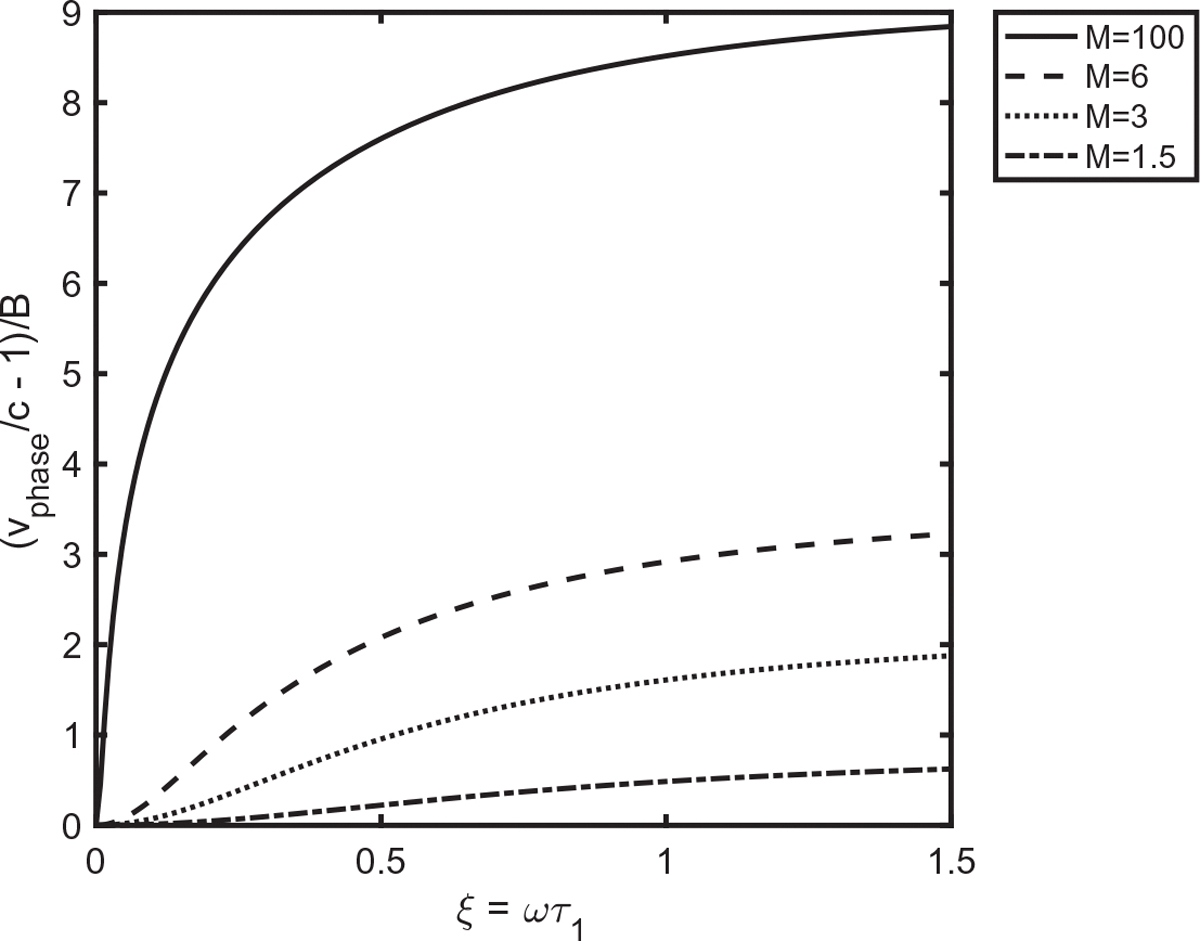
Plots of normalized phase velocity versus normalized frequency for multiple values of the parameter M.

Figure 2 gives plots of

(8.37) $$\frac{\left[\frac{v_{\text{phase}}}{c}\;-\;1\right]}{B}$$

versus ξ for M=1.5,M=3,M=6, and M=100 for 0<ξ<1.5. The parabolic behavior at small ξ is evident in the curves for the smaller values of M, but barely detectable for M=100. The upper limit of ξ=1.5 is sufficient for one to sense the limit where the function approaches a constant at large ξ.

## 9. Nonlinear Power Laws

For the generalized case when the exponent is a number a different from 1, the general expression for the integrals are

(9.1) $$\alpha =\frac{K_{a}\omega^{a}}{2c}\int_{\omega \tau_{1}}^{\omega \tau_{2}}\frac{\xi^{1-a}}{1\;+\;\xi^{2}}d\xi ,$$

(9.2) $$v_{ph}=c+\frac{c}{2}K_{a}\omega^{a-1}\int_{\omega \tau_{1}}^{\omega \tau_{2}}\frac{\xi^{2-a}}{1\;+\;\xi^{2}}d\xi .$$

In the low-frequency region where both $\omega \tau_{1}$ and $\omega \tau_{2}$ are substantially less than unity, one can expand the factor ${\left(1+\xi^{2}\right)}^{-1}$ in a power series in $\xi^{2}$ and then integrate term by term. Doing so yields

(9.3) $$\alpha \to K_{a}\frac{\omega^{2}}{2c}\frac{1}{2\;-\;a}\left(\tau_{2}^{2-a}-\tau_{1}^{2-a}\right),$$

(9.4) $$v_{ph}\to c+\frac{\omega^{2}c}{2}K_{a}\frac{1}{3\;-\;a}\left(\tau_{2}^{3-a}-\tau_{1}^{3-a}\right).$$

In a similar vein, in the high frequency region, one can expand the factor ${\left(1+\xi^{2}\right)}^{-1}$ in a power series in $\xi^{-2}$, and this yields

(9.5) $$\alpha \to \frac{K_{a}}{2c}\frac{1}{a}\left[\frac{1}{\tau_{1}^{a}}-\frac{1}{\tau_{2}^{a}}\right],$$

(9.6) $$v_{ph}\to c+\frac{c}{2}\frac{K_{a}}{a\;-\;1}\left[\frac{1}{\tau_{1}^{a-1}}-\frac{1}{\tau_{2}^{a-1}}\right].$$

The latter is indeterminate for the case (discussed above) of a=1. The appropriate replacement is

(9.7) $$\underset{a\to 1}{lim}\left\{\frac{1}{a\;-\;1}\left[\frac{1}{\tau_{1}^{a-1}}-\frac{1}{\tau_{2}^{a-1}}\right]\right\}=\ln\left(\frac{\tau_{2}}{\tau_{1}}\right).$$

For the attenuation in the mid-frequency region, where $1/\tau_{2}\ll \omega \ll 1/\tau_{1}$, it is a good approximation to replace the integration limits by 0 and ∞, and doing so yields

(9.8) $$\alpha \approx \frac{K_{a}\omega^{a}}{2c}I(a),$$

where

(9.9) $$I(a)=\int_{o}^{\infty }\frac{\xi^{1-a}d\xi }{1\;-\;\xi^{2}}=\frac{\frac{\pi }{2}}{\sin\frac{\pi a}{2}};\;0<a<2.$$

[The derivation of this integral is not necessarily trivial. Our derivation involved first expressing the integral as a beta function, then expressing the beta function in terms of gamma functions, then using the Euler reflection formula for the product of two gamma functions whose arguments sum to unity. The technique can be inferred from a monograph by Moll.^21^]

This mid-frequency expression for the attenuation confirms our expectation that α in this range should vary as $\omega^{a}$.

For general studies of the two integrals at arbitrary frequencies, it is convenient to express them in terms of a function J(u,v) of two variables where

(9.10) $$J\left(u,v\right)=\int_{o}^{v}\frac{\xi^{1-u}}{1\;+\;\xi^{2}}d\xi .$$

[The integral exists if u<2 and if v is finite.]

In terms of this function, we have

(9.11) $$\alpha =\frac{K_{a}\omega^{a}}{2c}\left[J\left(a,\omega \tau_{2}\right)-J\left(a,\omega \tau_{1}\right)\right],$$

(9.12) $$v_{ph}=c+\frac{c}{2}K_{a}\omega^{a-1}\left[J\left(a-1,\omega \tau_{2}\right)-J\left(a-1,\omega \tau_{1}\right)\right].$$

The function defined by Eq. (9.10) is simple enough to integrate numerically using standard software such as Matlab (and use of the trapezoidal rule with enough integration intervals might be sufficient), but it was decided, if at all possible, to express it in terms of another function that can be directly evaluated using available software packages. In this respect, it was found that J(u,v) could be expressed in terms of a hypergeometric function.^22^ A demonstration of the equivalence for small values of v begins with the expansion of the factor ${\left(1+\xi^{2}\right)}^{-1}$ in a power series

(9.13) $$\frac{1}{1\;+\;\xi^{2}}=1-\xi^{2}+\xi^{4}-\cdots =\sum_{n=0}^{\infty }{\left(-\xi^{2}\right)}^{n}.$$

The series converges only if $\xi^{2}<1$, so the proof that follows assumes that v<1. Insertion of this series into the integral and integration yields

(9.14) $$J(u,v)=\sum_{n=0}^{\infty }\frac{1}{2n\;+\;2\;-\;u}{\left(-1\right)}^{n}v^{2n+2-u}.$$

One can now attempt to relate this series to the hypergeometric series,

(9.15) F12(a,b;c;z)=1+abcz1!+a(a+1)b(b+1)c(c+1)z22!+⋯.

To achieve the correspondence, one writes

(9.16) $$J(u,v)=\frac{v^{2-u}}{2\;-\;u}\sum_{n=0}^{\infty }\frac{2\;-\;u}{2\;-\;u\;+\;2n}z^{n},$$

where

(9.17) $$z=-v^{2}.$$

If this is to yield a hypergeometric series, then one must have

(9.18) $$\frac{ab}{c}=\frac{2\;-\;u}{2\;-\;u\;+\;2};\;\frac{a(a\;+\;1)b(b\;+\;1)}{c(c\;+\;1)}\frac{1}{2!}=\frac{2\;-\;u}{2\;-\;u\;+\;4};\ldots$$

The factorials in the denominators are taken care of by setting a=1. Then, we can take care of the factorials involving b and c by setting c=b+1. So we have

(9.19) $$\frac{b}{b\;+\;1}=\frac{2\;-\;u}{2\;-\;u\;+\;2};\;\frac{b}{b\;+\;2}=\frac{2\;-\;u}{2\;-\;u\;+\;4};\ldots$$

Consequently, we identify

(9.20) $$a=1;\;b=1-\frac{u}{2};\;c=2-\frac{u}{2}.$$

and we can write

(9.21) J(u,v)=v2−u2−uF121,1−u2;2−u2;−v2.

Of possible concern is that the series definition for the hypergeometric function for the parameters as identified may not converge for v>1. However, the series representation is but one of many representations^23^ of the *hypergeometric function*, which can be broadly defined for all arguments. Analytic continuation leads to the supposition that the equivalence exists for the integral J(u,v) and the hypergeometric function, given that the latter is properly represented. Also, integral expressions for the hypergeometric function exist and, for the identified parameters, there may be no such problems. Using an integral representation of the hypergeometric function,^22^ we find

(9.22) F121,1−u2;2−u2;−v2=Γ2−u2Γ1−u2Γ1∫o1t−u2(1−tz)−1dt.

This expression, with the appropriate identities for the Gamma function, and when inserted into Eq. (9.21), leads back to our integral expression for J(u,v).

The Matlab routine that computes the hypergeometric function is named *hypergeom*.

Apart from explicitly calculating the functions numerically, it is of some value to seek simple explicit approximate expressions, as has been done in previous passages of this section. The phase velocity in the mid-frequency regime is cumbersome in general and depends critically on the value of a, so we would ordinarily prefer to simply use the general expression in terms of the function J(u,v). However, if 2>a>1, with a somewhat larger than 1, it may be an adequate approximation to also replace the limits by 0 and ∞. Then

(9.23) $$v_{ph}\approx c+\frac{c}{2}K_{a}\omega^{a-1}\int_{o}^{\infty }\frac{\xi^{2-a}}{1\;+\;\xi^{2}}d\xi .$$

The integral that appears here can also be evaluated using the technique alluded to above involving beta functions and gamma functions. The result is

(9.24) $$v_{ph}\approx c-\frac{c}{2}K_{a}\omega^{a-1}\frac{\frac{\pi }{2}}{\cos\frac{\pi a}{2}};\;2>a>1.$$

However, the approximation is clearly inappropriate if a=1 since cos(π/2)=0. For such a case, one must take into account that $\tau_{2}$ is finite.

## 10. Earlier Analytical Interpretations of Data

The observed dependence of attenuation and dispersion on frequency in natural media has long been a subject for potential interpretation in terms of distributed relaxation processes, so what is in the present paper is far from new in that respect. Most of the efforts have been for biological media, and we here briefly review some of the relevant prior work.

### 10.1. Carstensen and Schwan, 1959

Of especial note is the 1959 hemoglobin paper by Carstensen and Schwan,^12^ who used the idea of a continuous distribution of relaxation processes to explain an approximately linear frequency dependence of measured attenuation. They gave a suggested relation in Eq. (3),

(10.1) $$\alpha \lambda =B^{*}\omega +\int \;2(\alpha \lambda {)}^{*}f\left(\Omega \right)\frac{\frac{\omega }{\Omega }}{1\;+\;{\left(\frac{\omega }{\Omega }\right)}^{2}}d\Omega .$$

If we take λ as the nominal wavelength, with

(10.2) $$\lambda =\frac{2\pi c}{\omega },$$

then the first term in Carstensen and Schwan’s equation corresponds to the classical $\omega^{2}$ attenuation associated with, say, viscosity. We can also replace Ω by 1/τ, so their equation, for the relaxation part of the attenuation, becomes

(10.3) $$\alpha_{relax}=\int \;K\left(\tau \right)\frac{\omega^{2}}{1\;+\;{\left(\omega \tau \right)}^{2}}d\tau ,$$

where K(τ) is some function of τ. Comparison with our previously derived expression for attenuation yields

(10.4) $$K\left(\tau \right)=\frac{c}{2}\tau^{2}G\left(\tau \right).$$

The subsequent analysis regarding Eq. (3) was very similar to that given in the present paper for a=1. That assumed that f(Ω) was zero except for a region $\Omega_{1}<\Omega <\Omega_{2}$ and that, within this range, f(Ω) was of the form $\Omega_{o}/\Omega$. The appropriateness of this model was substantiated by a good fit with data in Fig. 8.

### 10.2. Other relevant earlier papers

Pauly and Schwan^24^ also used a continuous distribution of relaxation processes, combined as an integral over relaxation time constants, to explain measured attenuation in liver tissue. They proposed a power-law weighting function similar to that proposed here and evaluated the resulting absorption for several integer exponents.

In the context of interpreting measured attenuation in marine sediments, Pierce *et al.*^25^ proposed a weighted continuous distribution of relaxation processes, predicting approximately linear frequency dependence of attenuation over a wide frequency range, and derived associated dispersion relations.

Näsholm^26^ analyzed a power-law weighted distribution of relaxation processes over a finite bandwidth of relaxation frequencies, showing closed-form expressions for generalized compressibility that result in approximate power-law frequency dependence of attenuation over a finite frequency range, similar to results presented here.

A number of other investigators have proposed continuous distributions of relaxation processes with alternative weighting functions. For example, Choi *et al.*^27^ proposed a weight based on a mirrored Davidson–Cole function^28^ of the relaxation time. Berkhoff *et al.*^29^ employed a continuum of relaxation processes logarithmically distributed over a finite band of relaxation frequencies. Näsholm and Holm^30^ analyzed a distribution of relaxation processes based on the Mittag–Leffler function, demonstrating equivalence to a wave equation and dispersion relation based on a fractional Zener model. Any such weighting functions may potentially be analyzed by the methods applied here.

## 11. Data of Treeby, Zhang, Thomas, and Cox

**Fig. 3.**
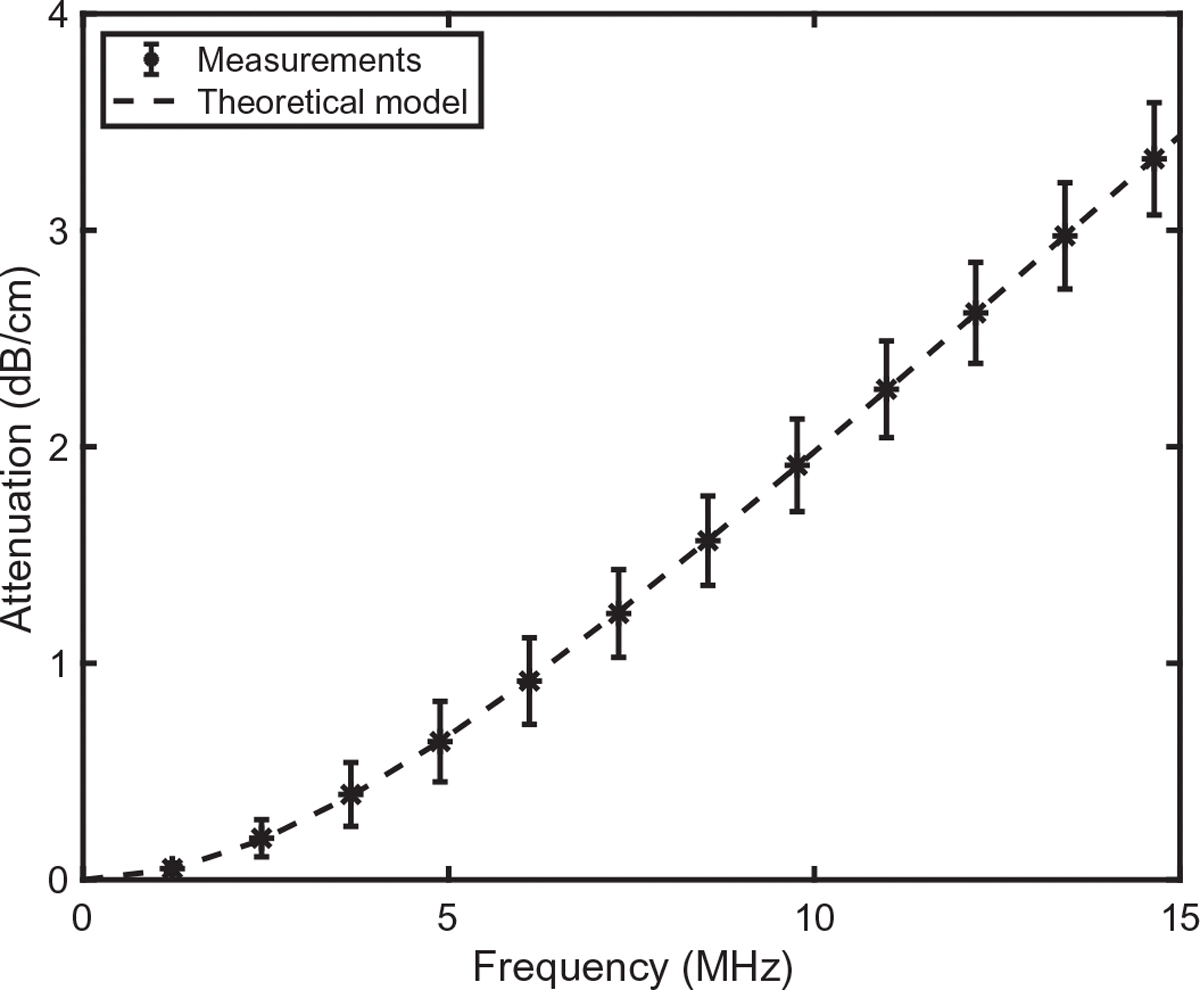
Comparison of measured attenuation with theoretical predictions of the present model. Measured data (mean ± standard deviation) is for a suspension of red blood cells in saline solution (total hemoglobin concentration 15mg/dL), as plotted in Fig. 3(c) of the paper by Treeby *et al.*^31^

There are many papers that present experimental plots of attenuation and phase velocity versus frequency in various media. The present authors believe that measured attenuation and phase velocity data can often largely be fitted with a judicious choice of the parameters of the model described in the previous section. One example of special significance is a paper on propagation through blood by Treeby *et al.*^31^ Their paper reviews a large body of work in this area and also presents significant new data. We chose to compare predictions from our theory with data plotted in Figs. 3(c) and 3(d). However, we are here using somewhat revised data corresponding to those figures that were communicated to us privately by Treeby.

The considered figures give plots of measured attenuation (dB/cm) and phase velocity (m/s) over the frequency range of 0–15 MHz. The measurements were at 32°C in solutions of human red cells in saline with a total hemoglobin concentration of 0.15 g/dL.

The measurements performed by Treeby and his colleagues covered the frequency range of 1–70 MHz, but our comparison with the data was restricted to the frequency range of 0–15 MHz. This was done so as to rule out any influence of scattering, and is in accord with the experimental observations of Shung *et al.*^32^ that acoustic attenuation due to scattering by blood is negligible within the frequency range less than 15 MHz. Treeby and colleagues suggested scattering contributions to attenuation may be comparable to those from relaxation at frequencies greater than 15 MHz. In addition, effects of viscous friction, resulting in attenuation proportional to the square of frequency, are expected to dominate relaxation effects at sufficiently high frequencies. Consistent with this observation, Treeby *et al.* found the frequency dependence of attenuation by blood to be different when fit over the range of 0–70 MHz, compared to 0–15 MHz.

Fits to measured data employed the present theoretical model with the weighting function $G(\tau )=K_{a}/\tau^{a}$ over the frequency range f<15MHz. Model fitting was performed by unconstrained nonlinear optimization using the *fminsearch* function in Matlab (R2019a, The MathWorks, Natick, MA). Phase velocity and attenuation were computed using Eqs. (9.11) and (9.12), respectively, with the integral J(u,v) numerically evaluated in terms of the hypergeometric function F12 by Eq. (9.21), using Matlab’s *hypergeom* function. This optimization returned values of the attenuation coefficient $K_{a}$, exponent a, relaxation-time bounds $\tau_{1}$ and $\tau_{2}$, and equilibrium sound speed c that minimized the summed root-mean-square (RMS) errors of attenuation and phase speed, normalized to measured RMS values.

Initial values provided to the *fminsearch* function were $\tau_{1}=1\;ns,\;\tau_{2}=100\;ns,\;c$ the measured phase speed extrapolated to zero frequency, a the power-law fit exponent determined by Treeby *et al*. over the frequency range f<70MHz, and $K_{a}$ the value matching measured mean attenuation over the fitted frequency range. Resulting optimized parameter values for red blood cells suspended in saline (total hemoglobin concentration 15g/dL) were $a=1.70,\;K_{a}=4.56\times 10^{-5}\mu {\;s}^{1-a},\;\tau_{1}=2.66\;ns,\;\tau_{2}=35.1\;ns$, and c=1575.4m/s.

**Fig. 4.**
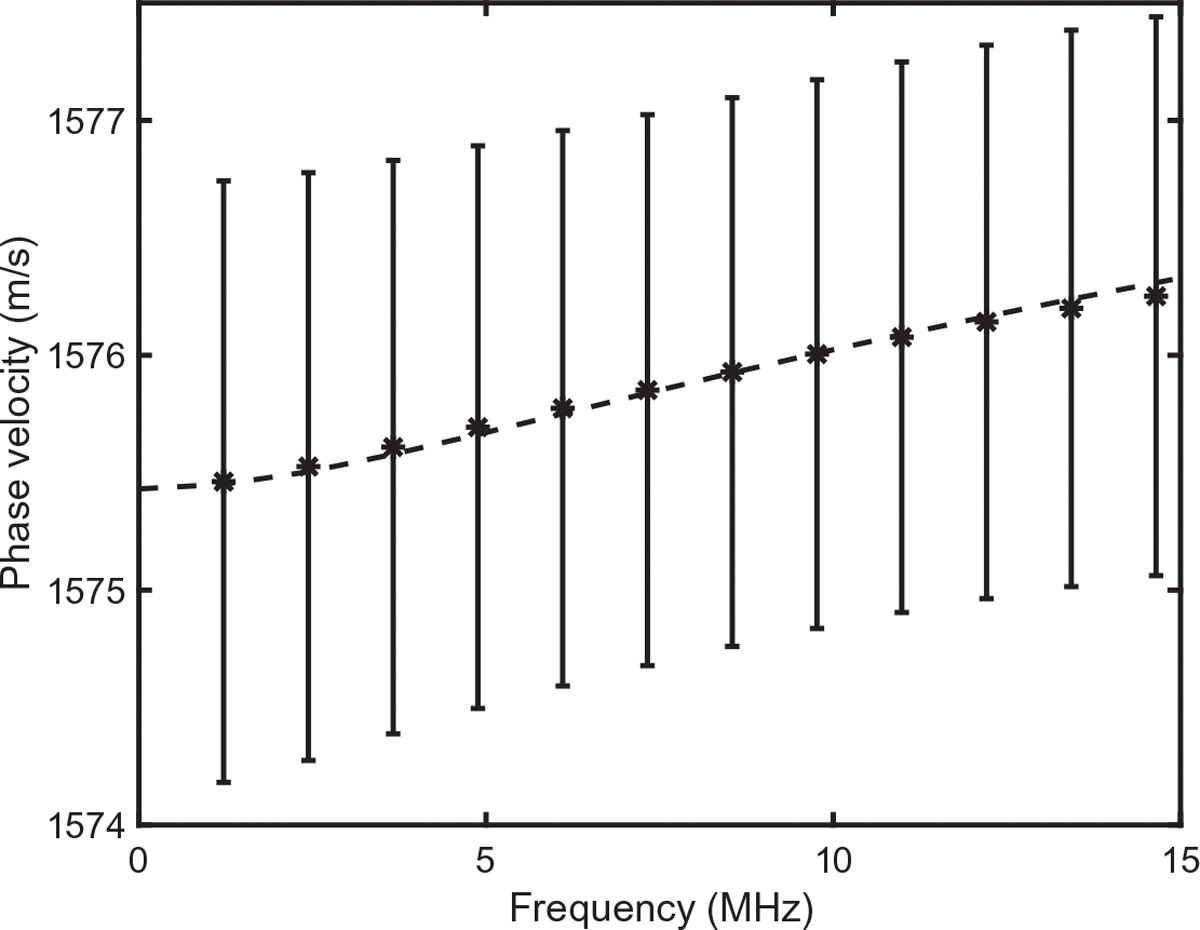
Comparison of measured attenuation with theoretical predictions of the present model. Measured data (mean ± standard deviation) is for a suspension of red blood cells in saline solution (total hemoglobin concentration 15 mg/dL), as plotted in Fig. 3(d) of the paper by Treeby *et al.*^31^

Resulting predictions are compared with measurements in Fig. 3 for attenuation and Fig. 4 for phase velocity. Attenuation in Fig. 3 is plotted in dB/cm, which may be converted to Np/m using the conversion factor

(11.1) $$1\;dB/cm=0.2\;{log}_{10}(e)\;Np/m.$$

The fits match measurements well, with normalized RMS errors < 0.3% for attenuation and < 0.002% for phase speed. Depending on initial values for fitted parameters, similarly good fits can also be obtained with somewhat different optimized values of $\tau_{1},\;\tau_{2}$, and a, suggesting that there are uncertainties inherent to estimating these parameters, and that dependencies on the parameters may be coupled. Thus, the fitted values found here should not be regarded as intrinsic properties of red blood cells. Comparison with Figures 3c and 3d in the paper by Treeby and colleagues [31] suggests that within the lower portion of the plotted frequency range, the present fit to attenuation is more accurate than a fit assuming power-law frequency dependence of attenuation at all frequencies.

## 12. Concluding Remarks

Relaxation processes are certainly not the only way sound can be attenuated in natural media, but may be the least understood. The present paper is largely conjectural, but one can easily conceive of media where there are a large number of possible relaxation processes. Small particles temporarily touching each other, as in clay clusters in mud, are an example. Local particles might be weakly held together by van der Waals forces, with bonds temporarily broken by the passage of a sound wave, then coming back together after the pressure returns to normal. Long chain molecules may have many local areas where the vibrational state can change abruptly when a sound wave passes by, and then it subsequently relaxes. It would seem that relaxation is a fundamental and nearly omnipresent process. The principal questions unanswered in the present paper concern just how strong are these processes and how are they distributed in their properties. Perhaps, the reported successes in comparison of experiment with theory may stimulate more concentrated research along those lines.

The authors argue that there may well be a large number of somewhat different processes with their own intrinsic relaxation times. General notions about the laws of large numbers support the conjecture that one can associate a relaxation strength function with the aggregate of relaxation process and that one can approximate this as a continuous function of relaxation times.

The general premises of the present paper are supported somewhat by the large number of experimental observations that suggest attenuation to vary with frequency over a large frequency range as a simple power law. As detailed in the present paper, distributed relaxation processes can explain this phenomenon. The authors do not know of any other fundamental process that can explain it.

While the amount of attenuation data existing in the literature is extremely large, the authors are encouraged that a model with a small number of adjustable parameters can explain the data of Treeby and his colleagues for propagation through a suspension of red blood cells. That the same model simultaneously explains both the attenuation versus frequency and the phase velocity versus frequency is of course mandated by the Kramers–Kronig relations, but it is nevertheless very encouraging that such works out to be the case.
